# Interaction of the *GCKR* and *A1CF* loci with alcohol consumption to influence the risk of gout

**DOI:** 10.1186/s13075-017-1369-y

**Published:** 2017-07-05

**Authors:** Humaira Rasheed, Lisa K. Stamp, Nicola Dalbeth, Tony R. Merriman

**Affiliations:** 1 Biochemistry Department, University of Otago, 710 Cumberland Street, Box 56, Dunedin, 9054 New Zealand; 2 University of Engineering and Technology, Lahore, Pakistan; 3 Department of Medicine, University of Otago, Christchurch, PO Box 4345, Christchurch, New Zealand; 4 Department of Medicine, University of Auckland, Auckland, New Zealand

## Abstract

**Background:**

Some gout-associated loci interact with dietary exposures to influence outcome. The aim of this study was to systematically investigate interactions between alcohol exposure and urate-associated loci in gout.

**Methods:**

A total of 2792 New Zealand European and Polynesian (Māori or Pacific) people with or without gout were genotyped for 29 urate-associated genetic variants and tested for a departure from multiplicative interaction with alcohol exposure in the risk of gout. Publicly available data from 6892 European subjects were used to test for a departure from multiplicative interaction between specific loci and alcohol exposure for the risk of hyperuricemia (HU). Multivariate adjusted logistic and linear regression was done, including an interaction term.

**Results:**

Interaction of any alcohol exposure with *GCKR* (*rs780094*) and *A1CF* (*rs10821905*) influenced the risk of gout in Europeans (interaction term 0.28, *P* = 1.5 × 10^−4^; interaction term 0.29, *P* = 1.4 × 10^−4^, respectively). At *A1CF*, alcohol exposure suppressed the gout risk conferred by the A-positive genotype. At *GCKR*, alcohol exposure eliminated the genetic effect on gout. In the Polynesian sample set, there was no experiment-wide evidence for interaction with alcohol in the risk of gout (all *P* > 8.6 × 10^−4^). However, at *GCKR*, there was nominal evidence for an interaction in a direction consistent the European observation (interaction term 0.62, *P* = 0.05). There was no evidence for an interaction of *A1CF* or *GCKR* with alcohol exposure in determining HU.

**Conclusions:**

These data support the hypothesis that alcohol influences the risk of gout via glucose and apolipoprotein metabolism. In the absence of alcohol exposure, genetic variants in the *GCKR* and *A1CF* genes have a stronger role in gout.

**Electronic supplementary material:**

The online version of this article (doi:10.1186/s13075-017-1369-y) contains supplementary material, which is available to authorized users.

## Background

Gout is an inflammatory arthritis that occurs in about one-fourth of people with elevated serum urate concentrations (hyperuricemia [HU]) [1] as a result of an innate immune system response to monosodium urate (MSU) crystals. Some ancestral groups exhibit a high prevalence of gout; for example, the Māori and Pacific (Polynesian) populations of New Zealand (NZ) have prevalence rates of 6% and 8%, respectively, compared with 3% among NZ Europeans [2]. A recent genome-wide association study (GWAS) of >140,000 European individuals identified 28 loci associated with urate concentration [3]. The loci with the strongest effects contain genes encoding uric acid transporters that regulate the excretion of uric acid. Not unexpectedly, most of the urate-raising alleles are also associated with increased risk of gout in various ancestral groups [3–5]. A prominent serum urate- and gout-associated locus in Oceanic populations is *LRP2* (encoding low-density lipoprotein receptor-related protein 2) [6–8].

Along with inherited genetic variants, the risk of gout and HU is influenced by dietary factors such as red meat, seafood, sugar-sweetened beverage, and alcohol consumption [8–14]. Earlier studies provided evidence that increased alcohol consumption is positively associated with the risk of HU [15–17] and gout [13, 18], with the association being stronger with beer consumption [8, 11]. The current literature posits that ethanol intake can contribute to HU, either by causing decreased renal excretion of uric acid [19, 20] that is secondary to lactate levels [17] whereby lactate can competitively inhibit urate secretion by the proximal tubule [21–23] and/or by increased urate production resulting from enhanced turnover of adenine dinucleotide phosphate as a result of hepatic processing of alcohol [16]. Regarding flares, more recent evidence from a prospective case-control crossover study suggests that alcohol exposure can act as a trigger for gout attacks. The close temporal association between alcohol exposure and a gout flare allows the hypothesis that alcohol plays a role in triggering the immune response to MSU crystals [24].

Potential gene–environment interactions are key features in the development of complex diseases, including gout and other rheumatic diseases such as rheumatoid arthritis (RA). An interaction occurs when the genetic factor and the environmental exposure interact in a way such that the combined effect does not reflect the independent individual effects. The finding of a gene–environment interaction between smoking and human leukocyte antigen DRB1 genotypes in patients with RA positive for antibodies to citrullinated proteins has led to significant knowledge on the etiology of RA [25, 26]. Similarly, evidence has been demonstrated for interaction of sugar-sweetened beverage consumption with a urate-associated variant of *SLC2A9* and alcohol intake with *LRP2* in determining the risk of gout and the risk of HU and gout, respectively [7, 8, 14]. The aim of the study we report here was to systematically test for a departure from multiplicative interaction between alcohol consumption and urate-associated loci in the risk of gout in people of European and Polynesian ancestry.

## Methods

### Participants

Cases (*n* = 1502) were recruited from rheumatology clinics, workplaces, and community focal points from the Auckland, Bay of Plenty, Wellington, Christchurch, and Dunedin areas of New Zealand. Control subjects with no self-reported history of gout (*n* = 1290) were enrolled as a convenience sample from workplaces and community focal points in the Auckland region of NZ. Gout status was clinically ascertained using the American College of Rheumatology criteria [27]. Recruitment occurred during the period 2006–2013. For analysis, subjects were divided into two ancestral groups: European (665 cases, 374 controls) and Polynesian (NZ Māori and Pacific Island people; 837 cases, 916 controls). The New Zealand Multi-Region Ethics Committee (MEC/105/10/130) granted ethical approval, and all participants gave written informed consent. The demographic and clinical information of participants is summarized in Table 1.

**Table 1:** Demographic and clinical data of study sample sets

	NZ European		NZ Polynesian		ARIC		FHS	
	Control	Gout	Control	Gout	NU	HU	NU	HU
Number	374	665	916	837	3443	690	2364	395
Male sex, *n* (%)	252 (67.4)	541 (81.3)	409 (44.8)	695 (83.0)	1255 (36.4)	581 (84.2)	878 (37.1)	376 (95.19)
Age, years	53.15 ± 17.02	63.13 ± 13.39	42.97 ± 15.08	51.75 ± 13.01	53.23 ± 5.49	53.49 ± 5.61	39.44 ± 8.72	39.38 ± 8.26
BMI, kg/m^2^	27.58 ± 6.11 (98.4)^a^	30.36 ± 5.56 (98.2)	33.03 ± 7.53 (98.4)	36.38 ± 8.25 (97.5)	25.41 ± 4.05 (100)	28.34 ± 4.16 (100)	25.71 ± 4.92 (100)	29.56 ± 4.80 (100)
Serum urate, mmol/L	0.335 ± 0.102 (93.6)	0.388 ± 0.113 (69.0)	0.375 ± 0.088 (83.2)	0.433 ± 0.115 (82.8)	0.305 ± 0.058 (100)	0.449 ± 0.041 (100)	0.281 ± 0.065 (100)	0.450 ± 0.040 (100)
Number of alcohol consumers, *n* (%)	277 (74.1)	463 (69.62)	373 (40.7)	365 (43.6)	1568 (45.5)	394 (57.1)	1881 (79.6)	328 (83.0)
Alcohol consumption, g/week	75.48 ± 96.36 (100)	80.77 ± 110.96 (100)	42.61 ± 91.41 (100)	54.91 ± 121.23 (100)	21.42 ± 42.94 (100)	36.56 ± 60.63 (100)	59.66 ± 79.27 (100)	99.35 ± 110.71 (100)
Triglycerides, mmol/L	1.74 ± 1.00 (93.3)	2.23 ± 1.47 (69.0)	2.02 ± 1.29 (83.2)	2.42 ± 1.67 (82.8)	1.26 ± 0.75 (99.9)	1.87 ± 1.07 (100)	2.58 ± 1.64 (100)	4.34 ± 3.82 (100)
eGFR, ml/min/1.73 m^2^	74.77 ± 16.85 (93.3)	62.41 ± 19.86 (68.1)	75.96 ± 16.73 (82.7)	64.37 ± 21.17 (81.6)	66.74 ± 9.65 (100)	65.83 ± 9.11 (100)	96.40 ± 16.94 (100)	93.89 ± 16.91 (100)

*Abbreviations: ARIC* Atherosclerosis Risk in Communities Study, *BMI* Body mass index, *eGFR* Estimated glomerular filtration rate, *FHS* Framingham Heart Study, *HU* Hyperuricemia, *NU* Normouricemia

All values are expressed as mean ± SD unless otherwise indicated

^a^For BMI, serum urate, triglyceride, eGFR, and alcohol consumption (g/week), the bracketed figure represents the percentage of subjects with data available

Data from the Framingham Heart Study (FHS; generation 3 only) and Atherosclerosis Risk in Communities Study (ARIC) cohorts were used for evaluating associations for risk of HU in Europeans. Subjects from the ARIC and FHS studies who self-reported as taking diuretic medication, who had a first-degree relative in the study, and who were not of European ancestry were excluded. Gout cases were also excluded. The ARIC dataset consisted of 4133 individuals and the FHS of 2759 individuals (Table 1).

### Data Collection

Serum urate concentrations were measured for NZ subjects by the uricase oxidation method, with the endpoint determined by using a Roche chemistry modular P/D analyzer (Roche Diagnostics, Indianapolis, IN, USA). In the ARIC dataset, serum urate was measured using the uricase oxidation method, and the endpoint was measured using a DACOS Chemistry Analyser (Coulter Electronics, Inc., Hialeah, FL, USA). In the FHS dataset, serum urate was measured using the carbonated method on an autoanalyzer with phosphotungstic acid reagent. On the basis of serum urate concentration, control participants in each study group were further stratified into a normouricemia group with serum urate concentration of <0.41 mmol/L and an HU group with a serum urate concentration ≥0.41 mmol/L. Estimated glomerular filtration rates (eGFRs) were derived from participants’ serum creatinine, age, and sex using the Chronic Kidney Disease Epidemiology Collaboration equation [28] for the NZ samples and the Modification of Diet in Renal Disease formula [29] for the ARIC and FHS samples.

For all study datasets, alcohol consumption was obtained by means of a food frequency questionnaire. NZ participants were asked at recruitment how many servings of beer, spirits, wine, and other alcohol they had consumed in the previous week. For ARIC, alcohol data were supplied as grams per day (from examination 1 in the years 1987–1989), and for FHS, the amount was provided as number of servings per week (beer, wine, and liquors) (from visit 1 for generation 3 in the years 2002–2005). The alcohol consumption data were converted into grams per week measurements in all datasets; details for conversion are described elsewhere [8].

### Genotyping

The lead associated single-nucleotide polymorphisms (SNPs) from 28 urate-associated loci [3] (*A1CF*, *ABCG2*, *ACVR1B/ACVRL1*, *ATXN2*, *MLXIPL/BAZ1B*, *GCKR*, *HLF*, *HNF4G*, *IGF1R*, *INHBB*, *INHBC*, *MAF*, *NFAT5*, *UBE2Q2*, *PDZK1*, *PRKAG2*, *RREB1*, *SFMBT1*, *SLC17A1*, *SLC22A11*, *SLC22A12*, *SLC16A9*, *SLC2A9*, *STC1*, *B3GNT4*, *TMEM171*, *TRIM46*, and *VEGFA*) were selected to test for interaction with alcohol consumption in determining the risk of gout. Additionally, *LRP2* was included as a sensitivity test on the basis of previously reported evidence for interaction of rs2544390 with alcohol consumption in determining the risk of gout in NZ Polynesian people [8].

The 29 SNPs were genotyped in the NZ sample set using either TaqMan SNP genotyping (Applied Biosystems, Foster City, CA, USA) or the MassARRAY System (Sequenom, San Diego, CA, USA) as described previously [4, 8]. The 29 SNPs were either imputed or genotyped in the ARIC and FHS sets. In the ARIC sample set, genotyping was performed using the Affymetrix SNP 6 platform (Affymetrix, Santa Clara, CA, USA), and imputation was conducted using IMPUTE version 2.0 for nongenotyped SNPs with reference to HapMap Centre d’Etude du Polymorphisme Humain/Utah residents with northern and western European ancestry (CEPH/CEU; National Center for Biotechnology Information build 37, version 37.3 [dbsnp135]). The FHS cohort had been genotyped using the Affymetrix SNP 5 platform and a custom-designed, gene-centric 50 K SNP platform, and data for nongenotyped variants were imputed using MACH1 version 1.0.15 with the HapMap2 CEPH/CEU (release 22, build 36) sample set as reference haplotypes [30].

### Statistical analysis

All analysis was done using Intercooled STATA™ version 13.0 software (StataCorp, College Station, TX, USA). An interaction term was calculated between alcohol consumption (as both a continuous and a binary variable) and genotyped SNPs (binary: individuals with at least one urate-raising allele [homozygous or heterozygous] and individuals homozygous for the urate-lowering allele) in linear and logistic regression analysis to assess the main effect association between gout/HU and alcohol consumption. All regressions were adjusted by age, sex, and BMI. Regression analysis of Polynesian samples was additionally adjusted for STRUCTURE ancestry and ancestral class. For STRUCTURE ancestry estimates, 67 biallelic genomic control markers had been genotyped, and STRUCTURE software [31] had previously been used to estimate the individual proportion of Polynesian ancestry [32]. A Bonferroni correction factor of 58 was applied to account for multiple testing, and a corrected *P* value <8.6 × 10^−4^ indicated statistical significance.

### Power calculation

The power of the study sample sets to detect an interaction for a range of allele frequencies in Europeans was calculated using Quanto version 1.2 (biostats.usc.edu/Quanto.html) for an uncorrected α = 8.6 × 10^−4^ (Additional file 1: Figure S1). The power for a given gene–environment interaction effect size was calculated using parameters that included prevalence rates of gout in the NZ population of 3% in Europeans and 7% in Polynesians [2], standardized regression coefficients relating the outcome (risk of gout) with the environmental exposure (alcohol consumption) (Re), tested gene (Rg), and the gene–environment interaction (Rge) with fixed minor allele frequencies in a recessive model.

## Results

### Interaction analysis for the risk of gout risk

Interaction terms for all 29 urate loci and alcohol consumption (as both continuous and binary exposure) for gout risk in the NZ European and Polynesian sample sets are presented in Table 2. In the European sample set, the variant rs780094 (*GCKR*) provided evidence for interaction with continuous alcohol consumption (interaction term 0.76, *P*_Interaction_ = 4.8 × 10^−4^) and variants rs10821905 (*A1CF*; interaction term 0.29, *P*_Interaction_ = 1.4 × 10^−4^) and rs780094 (*GCKR*; interaction term 0.28, *P*_Interaction_ = 1.5 × 10^−4^) for interaction with alcohol consumption as a binary variable (no alcohol intake vs. any alcohol intake). The interaction was independent of triglyceride levels and renal function (eGFR), with triglyceride- and eGFR-adjusted interaction terms of stronger effect for *GCKR* (interaction term 0.22, *P*_Interaction_ = 5.8 × 10^−5^; and 0.23, *P*_Interaction_ = 8.5 × 10^−5^, respectively) and a similar effect for *A1CF* (interaction term 0.28, *P*_Interaction_ = 6.3 × 10^−4^; and 0.29, *P*_Interaction_ = 1.0 × 10^−3^, respectively). At *GCKR*, nominal evidence in a consistent direction of association was seen in the Polynesian sample set for interaction with alcohol consumption as a binary variable (interaction term 0.62, *P* = 0.05), with the evidence strengthening after adjustment by triglyceride levels and eGFR (interaction term 0.54, *P*_Interaction_ = 0.02; and 0.53, *P*_Interaction_ = 0.02, respectively). However, there was no evidence for interaction of *A1CF* with alcohol consumption regarding risk of gout in the Polynesian sample set. Main effect association analysis of rs780094, rs10821905, and alcohol exposure in the risk of gout is presented in Additional file 1: Tables S1 and S2.

**Table 2:** Interaction terms between alcohol consumption and 29 urate loci for the risk of gout in New Zealand study cohorts

SNP (% genotype data)^a^	Gene value	Effect allele	NZ European (*n* = 1039)		NZ Polynesian (*n* = 1753)	
			Interaction term (SE)	*P* value	Interaction term (SE)	*P* value
Alcohol intake as a continuous variable (per 50 g/week)						
rs10821905 (99.2)	*A1CF*	A	0.85 (0.07)	0.026	1.09 (0.08)	0.28
rs2231142 (99.7)	*ABCG2*	T	0.99 (0.07)	0.89	0.92 (0.05)	0.19
rs7976059 (98.9)	*ACVR1B/ACVRL1*	T	0.95 (0.07)	0.44	0.94 (0.07)	0.33
rs653178 (99.3)	*ATXN2*	G	1.11 (0.07)	0.15	1.07 (0.09)	0.44
rs11983997 (97.8)	*MLXIPL/BAZ1B*	G	0.60 (0.22)	0.022	–	–
rs780094 (98.9)	*GCKR*	T	0.76 (0.08)	4.8 × 10^−4^	0.95 (0.06)	0.33
rs7224610 (99.2)	*HLF*	C	0.97 (0.07)	0.68	1.04 (0.06)	0.59
rs2941484 (98.9)	*HNF4G*	T	0.98 (0.07)	0.78	1.03 (0.06)	0.64
rs6598541 (99.0)	*IGF1R*	A	0.92 (0.08)	0.31	1.08 (0.16)	0.61
rs17050272 (99.0)	*INHBB*	A	0.99 (0.07)	0.96	0.98 (0.09)	0.78
rs1106766 (98.5)	*INHBC*	C	0.90 (0.48)	0.83	1.04 (0.17)	0.84
rs7188445 (98.8)	*MAF*	G	1.14 (0.14)	0.33	0.99 (0.08)	0.98
rs7193778 (98.8)	*NFAT5*	C	0.92 (0.08)	0.30	1.12 (0.07)	0.12
rs1394125 (98.6)	*UBE2Q2*	A	1.13 (0.07)	0.086	0.77 (0.12)	0.024
rs1967017 (99.0)	*PDZK1*	T	0.98 (0.08)	0.85	1.01 (0.10)	0.90
rs10480300 (98.0)	*PRKAG2*	T	0.95 (0.07)	0.45	1.17 (0.11)	0.16
rs675209 (99.2)	*RREB1*	T	0.83 (0.07)	0.007	1.08 (0.15)	0.61
rs6770152 (98.8)	*SFMBT1*	G	0.98 (0.07)	0.81	0.97 (0.07)	0.72
rs1183201 (99.5)	*SLC17A1*	T	0.96 (0.10)	0.73	1.01 (0.10)	0.91
rs2078267 (99.7)	*SLC22A11*	G	0.98 (0.08)	0.86	1.00 (0.22)	1.00
rs3825018 (99.7)	*SLC22A12*	G	0.89 (0.06)	0.11	1.05 (0.11)	0.67
rs12356193 (98.3)	*SLC16A9*	A	1.07 (0.29)	0.81	–	–
rs11942223 (99.9)	*SLC2A9*	T	0.96 (0.21)	0.85	0.52 (2.74)	0.81
rs17786744 (99.0)	*STC1*	G	1.08 (0.07)	0.29	1.11 (0.06)	0.055
rs7953704 (99.2)	*B3GNT4*	G	0.99 (0.09)	0.90	1.04 (0.07)	0.52
rs17632159 (98.6)	*TMEM171*	G	1.25 (0.14)	0.10	0.86 (0.11)	0.16
rs11264341 (99.1)	*TRIM46*	C	1.01 (0.09)	0.95	1.03 (0.06)	0.63
rs729761 (99.1)	*VEGFA*	G	1.11 (0.14)	0.46	0.99 (0.10)	0.98
rs2544390 (79.4)	*LRP2*	T	0.92 (0.09)	0.34	0.72 (0.11)	0.004
Alcohol intake as a categorical variable (none vs. any intake)						
rs10821905 (99.2)	*A1CF*	A	0.29 (0.10) 0.28 (0.10)^b^ 0.29 (0.11)^c^	1.4 × 10^−4^ 6.3 × 10^−4^ 1.0 × 10^−3^	1.27 (0.36)	0.40
rs2231142 (99.7)	*ABCG2*	T	1.05 (0.38)	0.88	0.91 (0.23)	0.71
rs7976059 (98.9)	*ACVR1B/ACVRL1*	T	0.75 (0.24)	0.38	0.72 (0.19)	0.22
rs653178 (99.3)	*ATXN2*	G	1.90 (0.70)	0.082	0.91 (0.79)	0.79
rs11983997 (97.8)	*MLXIPL/BAZ1B*	G	0.19 (0.19)	0.093	–	–
rs780094 (98.9)	*GCKR*	T	0.28 (0.09) 0.22 (0.08)^b^ 0.23 (0.09)^c^	1.5 × 10^−4^ 5.8 × 10^−5^8.5 × 10^−5^	0.62 (0.15) 0.54 (0.14) 0.53 (0.14)	0.05 0.02 0.02
rs7224610 (99.2)	*HLF*	C	0.77 (0.25)	0.43	1.26 (0.34)	0.38
rs2941484 (98.9)	*HNF4G*	T	1.79 (0.62)	0.092	1.36 (0.21)	0.21
rs6598541 (99.0)	*IGF1R*	A	1.21 (0.40)	0.57	1.32 (0.84)	0.66
rs17050272 (99.0)	*INHBB*	A	0.49 (0.16)	0.033	1.32 (0.46)	0.43
rs1106766 (98.5)	*INHBC*	C	0.52 (0.36)	0.35	1.54 (0.86)	0.86
rs7188445 (98.8)	*MAF*	G	1.92 (1.07)	0.24	0.98 (0.32)	0.95
rs7193778 (98.8)	*NFAT5*	C	0.63 (0.23)	0.20	1.11 (0.31)	0.70
rs1394125 (98.6)	*UBE2Q2*	A	2.38 (0.79)	0.009	0.63 (0.22)	0.18
rs1967017 (99.0)	*PDZK1*	T	0.83 (0.30)	0.60	1.26 (0.48)	0.54
rs10480300 (98.0)	*PRKAG2*	T	1.07 (0.34)	0.83	1.01 (0.42)	0.97
rs675209 (99.2)	*RREB1*	T	0.68 (0.22)	0.23	1.33 (0.75)	0.61
rs6770152 (98.8)	*SFMBT1*	G	1.26 (0.44)	0.51	1.07 (0.32)	0.82
rs1183201 (99.5)	*SLC17A1*	T	0.53 (0.22)	0.12	1.80 (0.83)	0.20
rs2078267 (99.7)	*SLC22A11*	G	1.20 (0.42)	0.60	0.60 (0.55)	0.58
rs3825018 (99.7)	*SLC22A12*	G	1.27 (0.40)	0.45	1.00 (0.41)	0.99
rs12356193 (98.3)	*SLC16A9*	A	1.03 (0.99)	0.97	–	–
rs11942223 (99.9)	*SLC2A9*	T	0.83 (0.71)	0.82	1.12 (2.96)	0.97
rs17786744 (99.0)	*STC1*	G	1.00 (0.34)	0.99	1.72 (0.43)	0.03
rs7953704 (99.2)	*B3GNT4*	G	1.28 (0.52)	0.54	0.68 (0.24)	0.27
rs17632159 (98.6)	*TMEM171*	G	1.71 (1.02)	0.37	0.38 (0.16)	0.022
rs11264341 (99.1)	*TRIM46*	C	1.45 (0.59)	0.36	1.07 (0.27)	0.80
rs729761 (99.1)	*VEGFA*	G	1.81 (0.97)	0.26	0.99 (0.41)	0.97
rs2544390 (79.4)	*LRP2*	T	0.86 (0.34)	0.71	0.32 (0.12)	0.003

*NZ* New Zealand, *SNP* Single-nucleotide polymorphism

Regression analyses are adjusted for age, sex, and body mass index. The Polynesian analyses are additionally adjusted for STRUCTURE ancestry and ancestral class (Western vs. Eastern Polynesian vs. mixed Western/Eastern Polynesians). No data are presented for rs11983997 and rs12356193 in people of Polynesian ancestry, owing to the minor genotype group having very low frequency (<0.01)

^a^For each individual SNP, the number inside the parentheses represents the percentage of subjects with available genotype data

^b^The interaction term was additionally adjusted for triglycerides (mmol/L)

^c^The interaction term was additionally adjusted for estimated glomerular filtration rate (ml/minute/1.73 m^2^)

### Interaction analysis for risk of HU

*GCKR* and *A1CF* were specifically tested for interaction with alcohol in determining the risk of HU. There was no evidence for interaction of *A1CF* (interaction term 0.76, *P*_Interaction_ = 0.25) and *GCKR* (interaction term 0.90, *P*_Interaction_ = 0.54) with binarized alcohol consumption for the risk of HU in the combined ARIC and FHS European cohorts. Additional adjustment for triglycerides and eGFR did not substantively change the interaction terms (all *P* > 0.13).

### Association analysis in stratified groups

To understand the nature of the interaction with alcohol consumption, adjusted ORs in the separate genotype groups were calculated for the risk of gout in Europeans, using alcohol as a binary variable (Table 3). At *A1CF*, alcohol exposure suppressed the gout risk conferred by the A-positive genotype (Table 3) (OR 2.21, *P* = 0.007 in the unexposed; vs. OR 0.93, *P* = 0.76 in the exposed group). At *GCKR*, alcohol exposure eliminated the genetic effect of rs780094 on the risk of gout: In the alcohol-exposed group, the ORs for the T-negative and T-positive genotype groups were 2.07 and 2.39, respectively, and were not significantly different (OR for T-positive alcohol-exposed group vs. the T-negative alcohol-exposed group was 1.12, *P* = 0.51). In contrast, the OR for the T-positive group in the alcohol nonexposed group compared with T-negative group was 4.13 (*P* = 8.7 × 10^−7^). A similar pattern was observed in the Polynesian sample set (Table 4): The OR for T positivity in the alcohol nonexposed group compared with T negativity was 1.92 (*P* = 3.2 × 10^−5^), and in the exposed group the equivalent OR was 1.24 (*P* = 0.25).

**Table 3:** Alcohol intake and gout association results in genotype-stratified groups in people of European ancestry for *A1CF* and *GCKR*

	No alcohol intake (*n* = 297)		Any alcohol intake (*n* = 734)		
	Adjusted OR (95% CI)	*P* value	Adjusted OR (95% CI)	*P* value	*P*_Difference_
*A1CF*					
A−	1	1	1.47 (0.99–2.16)	0.054	
A+	2.21 (1.24–3.93)	0.007	0.93 (0.61–1.43)	0.76	0.011
Beer					
A−	1	1	1.66 (1.13–2.43)	0.009	
A+	1.42 (0.96–2.10)	0.077	0.79 (0.51–1.23)	0.30	0.002
Nonbeer					
A−	1	1	0.97 (0.68–1.39)	0.89	
A+	1.48 (0.92–2.39)	0.10	0.63 (0.42–0.94)	0.023	0.019
	No alcohol intake (*n* = 296)		Any alcohol intake (*n* = 727)		
*GCKR*					
T−	1	1	2.07 (1.24–3.47)	0.0050	
T+	4.13 (2.35–7.26)	8.65 × 10^−7^	2.39 (1.47–7.26)	0.00043	0.51
Beer					
T−	1	1	1.57 (0.96–2.55)	0.071	
T+	2.04 (1.39–3.00)	0.0003	1.87 (1.22–2.88)	0.004	0.54
Nonbeer					
T−	1	1	1.62 (1.01–2.59)	0.046	
T+	3.56 (2.23–5.68)	9.82 × 10^−8^	1.57 (1.03–2.40)	0.037	0.77

Associations are adjusted for age, sex, and body mass index. *P*_Difference_ is that between genotype groups in the alcohol-exposed subset

**Table 4:** Alcohol intake and gout association results in genotype-stratified groups in people of Polynesian ancestry for *GCKR*

		No alcohol intake (*n* = 1007)		Any alcohol intake (*n* = 732)		
		Adjusted OR (95% CI)	*P* value	Adjusted OR (95% CI)	*P* value	*P*_Difference_
All	T-	1	1	1.73 (1.21–2.46)	0.002	
	T+	1.92 (1.41–2.62)	3.18 × 10^−5^	2.07 (1.47–2.92)	2.91 × 10^−5^	0.25
Beer	T−	1	1	1.60 (1.10–2.32)	0.013	
	T+	1.70 (1.28–2.25)	2 × 10^−4^	2.17 (1.50–3.13)	3.79 × 10^−5^	0.13
Nonbeer	T−	1	1	1.68 (1.09–2.59)	0.020	
	T+	1.78 (1.37–2.32)	1.95 × 10^−5^	1.81 (1.22–2.67)	0.003	0.66

Associations are adjusted for age, sex, body mass index, STRUCTURE ancestry estimates, and ancestry class (Western vs. Eastern Polynesian vs. mixed Western/Eastern Polynesians). *P*_Difference_ is that between genotype groups in the alcohol-exposed subset

### Stratification by type of alcohol

Given epidemiological data suggesting a stronger role for beer in the risk of gout [8, 13], we tested for interaction, stratifying by beer vs. nonbeer exposure at *A1CF* and *GCKR* in Europeans. There was no evidence for a differential interaction with *A1CF* (interaction term 0.46, *P* = 0.003 in the beer-exposed group; vs. interaction term 0.46, *P* = 0.007 in the non-beer-exposed group), whereas only the non-beer-exposed group exhibited statistically significant evidence for interaction at *GCKR* (interaction term 0.58, *P* = 0.079 in the beer-exposed group; vs. interaction term 0.27, *P* = 2.6 × 10^−5^ in the non-beer-exposed group). Calculation of ORs for beer and nonbeer exposure in the genotype groups revealed a similar pattern to that seen in the all alcohol group (Tables 3 and 4).

## Discussion

In this study, interaction of urate loci with alcohol consumption in determining the risk of gout was systematically evaluated. Significant interaction (*P*_corrected_ < 0.05) was observed with *A1CF* and *GCKR* in the European sample set, with nominal evidence for interaction with *GCKR* in the Polynesian sample set with a consistent direction of effect (Table 2). At both loci, visualization in genotype-stratified groups revealed that alcohol exposure negated or fully suppressed the genetic effect (Table 3). This is consistent with inherited genetic variation at each of these loci contributing to the risk of gout through a particular etiological pathway shared with alcohol exposure (discussed below), with this genetic risk being less important upon exposure to alcohol.

It is noteworthy that none of the urate transporter loci (*SLC2A9*, *SLC17A1*, *SLC22A11*, *SLC22A12*, *ABCG2*) showed any evidence for interaction with alcohol consumption in any of the study sample sets. Rather, the loci (*GCKR* and *A1CF*) interacting with alcohol consumption in determining the risk of gout in Europeans are involved in glycolysis and apolipoprotein metabolism, respectively. This suggests that alcohol could act on gout risk through metabolic pathways in addition to or instead of directly interfering with renal or extrarenal uric acid excretion. Presumably, the urate-raising and gout risk allele of *GCKR* results in increased production of gout-causing metabolites through glycolysis. Upon exposure to alcohol, however, individuals with the risk allele no longer exhibit increased metabolite production (compared with those with the protective allele), owing to alcohol “saturating” the causal pathway and eliminating the genetic discrimination seen in the absence of alcohol. In the case of *A1CF*, it can be speculated that acetate, the end product of alcohol oxidation, is a precursor for acyl-coenzyme A (CoA), an intermediate needed for the production of diacylglycerol CoA. Activation of protein kinase C via diacylglycerol-CoA leads to the phosphorylation and localization of *A1CF* in the nucleus and thus causes overproduction of apolipoprotein B-48 (ApoB-48) due to excessive editing of ApoB messenger RNA (mRNA). The consequent lower production of ApoB-100 might be responsible for more availability/hydrolysis of very low-density lipoprotein (VLDL) triglyceride and thus more production of free fatty acids to synergize the immunogenic response from MSU crystals. Previously, we reported an association of triglycerides, and in particular VLDL triglycerides, with gout [33], as well as an association of a triglyceride-decreasing apolipoprotein C3 (*APOC3*) variant (rs5128) with decreased risk of gout [34]. However, the interactions reported here were independent of triglyceride levels (Tables 2, 3 and 4). It would be useful to evaluate any possible influence of VLDL triglyceride on the interactions; however, we do not have these data for the cohorts studied here.

The apolipoprotein B mRNA editing enzyme, catalytic polypeptide 1, complementation factor (A1CF), protein is known as a complementation factor for the apolipoprotein B mRNA editing enzyme, catalytic polypeptide 1 (APOBEC1), complex and thus plays a preliminary role in the production of ApoB (both isoforms ApoB-48 and ApoB-100). High levels of VLDL triglyceride and its associated ApoB have been observed in gout [33]. The presence of ApoB assists in oxidative degradation of triglyceride-rich lipoproteins, including VLDL and low-density lipoprotein (LDL), that results in the endogenous production of free fatty acids. Members of the fatty acid family exert inflammatory effects via stimulation of the Toll-like receptor 2 (TLR2) and TLR4 signaling pathways [35], along with activation of the nucleotide-binding domain leucine-rich family pyrin domains-containing 3 (NLRP3) inflammasome that is a molecular platform for the processing and release of interleukin (IL)-1β [36]. Joosten et al. [37] provided experimental evidence for the essential involvement of free fatty acids as a stimulatory signal, along with MSU crystal for the NLRP3 inflammasome-mediated production of IL-1β. These findings are further supported by another study [38] where the researchers observed that, along with MSU crystals, acetate is necessary for adequate production of IL-1β in a murine model of gout. Thus, the production of free fatty acids, either from food (e.g., alcohol oxidized to acetate) or from an endogenous source, can potentiate the effect of MSU crystal-induced gouty inflammation. It is important to note, however, that the *A1CF* interaction was not replicated in the Polynesian sample set. Acknowledging that the alcohol–*AICF* interaction in Europeans could be a false-positive finding, lack of replication could be due to insufficient power and/or different biological pathways through which alcohol influences gout.

One important caveat in interpreting the biological significance of these findings is the assumption of whether the GWAS-identified *GCKR* and *A1CF* genes are causal. As reviewed previously [39], the urate association signals at each locus often extend over more than one gene; thus, multiple candidate genes can exist. Referring to the local association patterns as reported by Köttgen et al. [3] (Additional file 1: Figure S2), *A1CF* maps underneath a clearly defined peak of association. At the *GCKR* locus, however, there are a number of genes under the region of association, although *GCKR* contains a maximally associated candidate nonsynonymous causal variant (rs1260326; P446L; in very strong linkage disequilibrium with rs780094).

There are several limitations of this study. First, the sample sets were relatively small and had limited power. The *GCKR* finding did replicate between the European and Polynesian sample sets; however, it is possible that there are undetected interactions (i.e., false-negative results). Certainly, the analysis should be repeated in a larger sample set with the aim of replicating the *GCKR* and *A1CF* findings and detecting additional interactions with alcohol exposure. Second, the gout case-control sample sets were drawn from the population of New Zealand. This limits the generalizability to other countries and further emphasizes the need to replicate and extend these findings elsewhere. Finally, the self-reported nature of the subjects’ alcohol consumption data used here might have influenced the results to some extent. There is evidence to suggest that alcohol consumption data could be adversely affected by bias from underreporting and thus could affect the validity of measures of consumption of alcohol [40, 41].

## Conclusions

This systematic analysis provides the first evidence for an interaction of alcohol consumption (an established dietary risk factor for gout) with previously identified urate/gout-associated loci *GCKR* and *A1CF* that are predominantly involved in glycolysis and lipid homeostasis. None of the urate transporter loci showed significant interactions with alcohol consumption. Our findings are consistent with alcohol consumption causally contributing to gout via glycolysis and apolipoprotein metabolism. In the absence of alcohol exposure, genetic variants in the *GCKR* and *A1CF* genes have a stronger role in determining the risk of gout.

## Additional file

Additional file 1:
**Tables S1** and **S2** and **Figures S1** and **S2**. (DOCX 3399 kb)

## Abbreviations

*A1CF*: Apolipoprotein B mRNA editing enzyme, catalytic polypeptide 1, complementation factor gene
*ABCG2*: Adenosine triphosphate-binding cassette G2 gene
ApoB: Apolipoprotein B
APOBEC1: Apolipoprotein B mRNA editing enzyme, catalytic polypeptide 1
*APOC3*: Apolipoprotein C3 gene
ARIC: Atherosclerosis Risk in Communities Study
BMI: Body mass index
CEPH/CEU: Centre d’Etude du Polymorphisme Humain/Utah residents with northern and western European ancestry
CoA: Coenzyme A
DACOS: Discrete Analyser with Continuous Optical Scanning
eGFR: Estimated glomerular filtration rate
FHS: Framingham Heart Study
*GCKR*: Glucokinase regulatory protein gene
GWAS: Genome-wide association study
HU: Hyperuricemia
IL: Interleukin
LDL: Low-density lipoprotein
*LRP2*: Low-density lipoprotein-related protein 2 gene
mRNA: Messenger RNA
MSU: Monosodium urate
NLRP3: Nucleotide-binding domain leucine-rich family pyrin domains-containing 3
NU: Normouricemia
NZ: New Zealand
RA: Rheumatoid arthritis
*SLC17A1*: Solute carrier family 17, member 1 gene
*SLC22A11*: Solute carrier family 22, member 11 gene
*SLC22A12*: Solute carrier family 22, member 12 gene
*SLC2A9*: Solute carrier family 2, member 9 gene
SNP: Single-nucleotide polymorphism
TLR: Toll-like receptor
VLDL: Very low-density lipoprotein
