# Supplementary material for: Interaction of the GCKR and A1CF loci with alcohol consumption to influence the risk of gout
Source: Arthritis Res Ther. 2017 Jul 5;19:161. doi: 10.1186/s13075-017-1369-y (PMC5499049; doi:10.1186/s13075-017-1369-y)
Supplement: Additional file 1: — Tables S1 and S2 and Figures S1 and S2. (DOCX 3399 kb) [file 13075_2017_1369_MOESM1_ESM.docx]

## SUPPLEMENTAL MATERIAL

## Interaction of the *GCKR* and *A1CF* loci with alcohol consumption to influence the risk of gout

Humaira Rasheed^1,2^, Lisa K Stamp^3^, Nicola Dalbeth^4^, Tony R Merriman*^1^

1, Department of Biochemistry, University of Otago, Box 56, Dunedin, New Zealand; 2, University of Engineering and Technology, Lahore, Pakistan; 3, Department of Medicine, University of Otago, Christchurch, PO Box 4345, Christchurch, New Zealand; 4, Department of Medicine, University of Auckland, Auckland, New Zealand.

Email addresses: [rasheed.humaira@gmail.com](mailto:rasheed.humaira@gmail.com); [lisa.stamp@cdhb.health.nz](mailto:lisa.stamp@cdhb.health.nz); [n.dalbeth@auckland.ac.nz](mailto:n.dalbeth@auckland.ac.nz); tony.merriman@otago.ac.nz

*Corresponding author: Biochemistry Department, 710 Cumberland Street, University of Otago, Dunedin 9054, New Zealand.

Table S1: Association analysis of *A1CF* and *GCKR* with the risk of gout

|  | Case Genotypes, n (freq) | | | | Control Genotypes, n (freq) | | | | Unadjusted |  | Adjusted^1^ |  |
| --- | --- | --- | --- | --- | --- | --- | --- | --- | --- | --- | --- | --- |
| ***Rs10821905* (*A1CF*)** | | | | | | | | | | |  |  |
|  | GG | GA | AA | A Freq | GG | GA | AA | A Freq | Allelic OR (A-Allele), [95% CI] | Allelic P | Allelic OR (A-Allele), [95% CI] | Allelic P |
| European | 433(0.659) | 193(0.294) | 31(0.047) | 255(0.194) | 238(0.636) | 125(0.334) | 11(0.029) | 147(0.196) | 0.98[0.79-1.23] | 0.89 | 1.01[0.79-1.29] | 0.91 |
| Polynesians | 646(0.779) | 174(0.201) | 09(0.011) | 192(0.116) | 695(0.765) | 198(0.212) | 16(0.018) | 230(0.126) | 0.90[0.73-1.11] | 0.33 | 1.07[0.83-1.37] | 0.61 |
| ***Rs780094* (*GCKR*)** | | | | | | | | | | |  |  |
|  | CC | CT | TT | T Freq | CC | CT | TT | T Freq | Allelic OR (T-Allele), [95% CI] | Allelic P | Allelic OR (T-Allele), [95% CI] | Allelic P |
| European | 206(0.317) | 313(0.482) | 130(0.200) | 573(0.441) | 154(0.412) | 168(0.449) | 52(0.139) | 272(0.364) | 1.37[1.14-1.65] | 0.001 | 1.48[1.21-1.81] | 0.0001 |
| Polynesian | 358(0.433) | 347(0.420) | 121(0.146) | 589(0.356) | 471(0.516) | 359(0.393) | 83(0.091) | 525(0.287) | 1.35[1.17-1.55] | 2.67x10^-5^ | 1.48[1.25-1.76] | 7.46x10^-6^ |

Associations are adjusted for age, sex and BMI. The Polynesian sample set is additionally adjusted for STRUCTURE ancestry estimates and ancestry class (Western vs Eastern Polynesian vs mixed Western/Eastern Polynesians).

**Table S2.** Alcohol intake (none vs any alcohol intake) and risk of gout

|  | **Unadj OR[95% CI]** | **P** | **Adj OR[95% CI]** | **P** |
| --- | --- | --- | --- | --- |
| Gout | | | | |
| All Alcohol Types |  |  |  |  |
| NZ European | 0.80[0.60-1.07] | 0.13 | 0.95[0.69-1.30] | 0.76 |
| Polynesian | 1.12[0.93-1.36] | 0.22 | 1.35[1.06-1.72] | 0.017 |
| Beer |  |  |  |  |
| NZ European | 1.13[0.87-1.47] | 0.36 | 1.11[0.81-1.51] | 0.51 |
| Polynesian | 1.67[1.35-2.05] | 1.43x10^-6^ | 1.40[1.08-1.82] | 0.011 |
| Non-Beer Alcohol |  |  |  |  |
| NZ European | 0.65[0.50-0.84] | 0.001 | 0.73[0.55-0.98] | 0.036 |
| Polynesian | 0.72[0.58-0.90] | 0.005 | 1.29[0.96-1.72] | 0.086 |

Associations are adjusted for age, sex, BMI and, for Polynesian analyses, STRUCTURE ancestry estimates and ancestry class (Western vs Eastern Polynesian and Mixed Western Eastern Polynesians).

**Figure S1**
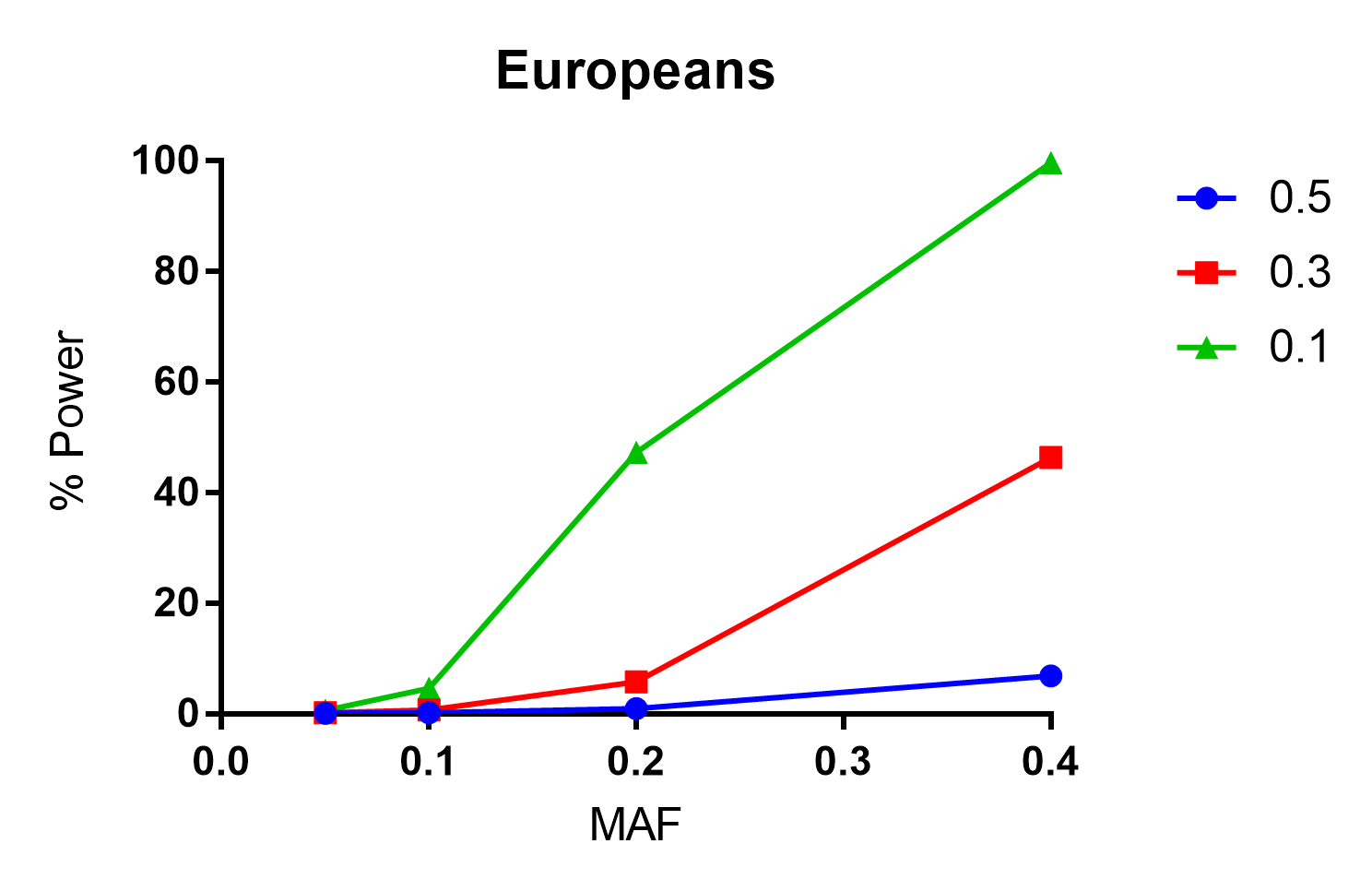

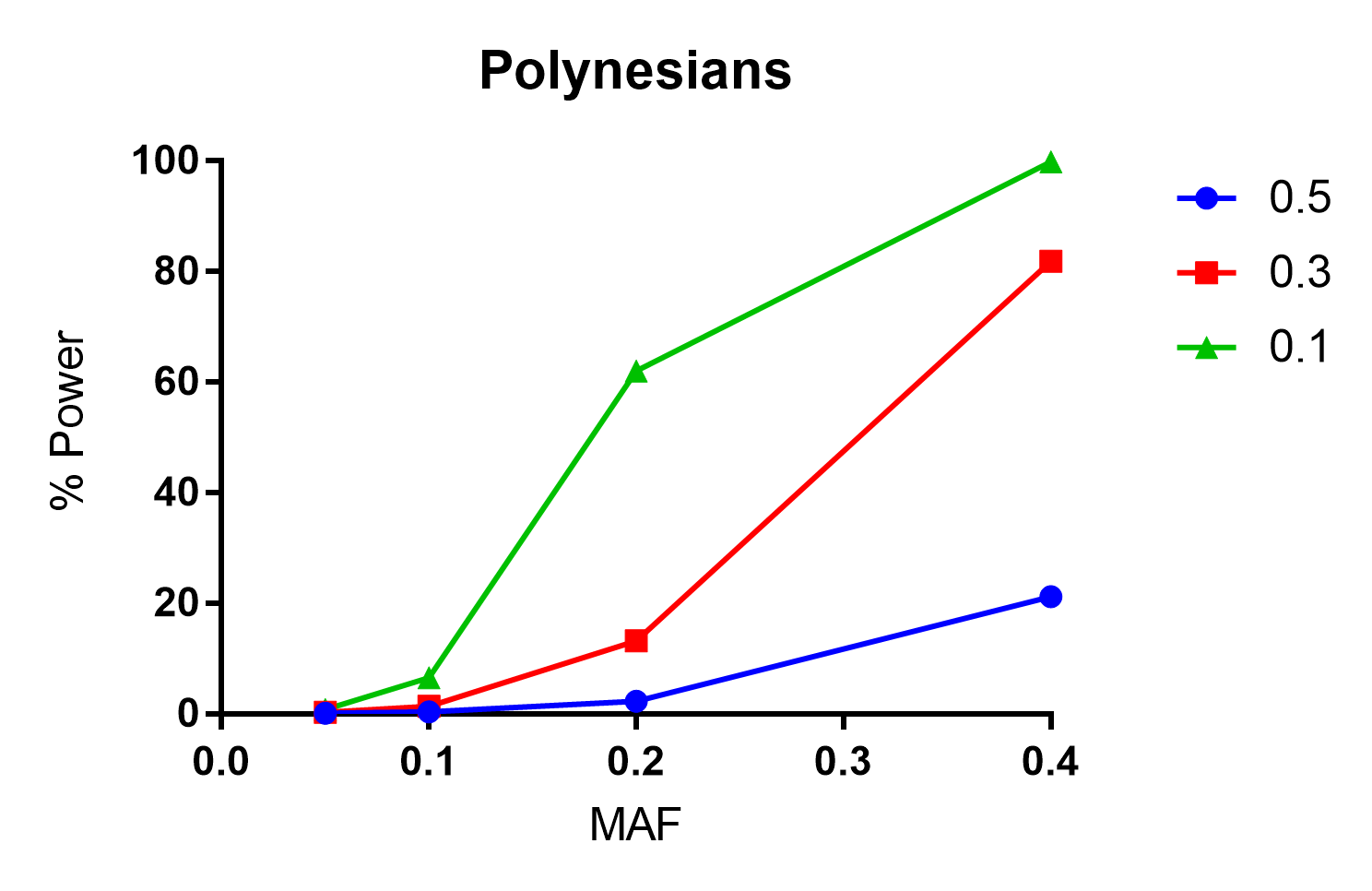


**Figure S1.** Power calculation in the study sample sets to detect gene-environment interaction effect sizes of 0.1, 0.3 and 0.5 with α=8.6×10^-4^ for various minor allele frequencies (MAF).

**Figure S2**

| 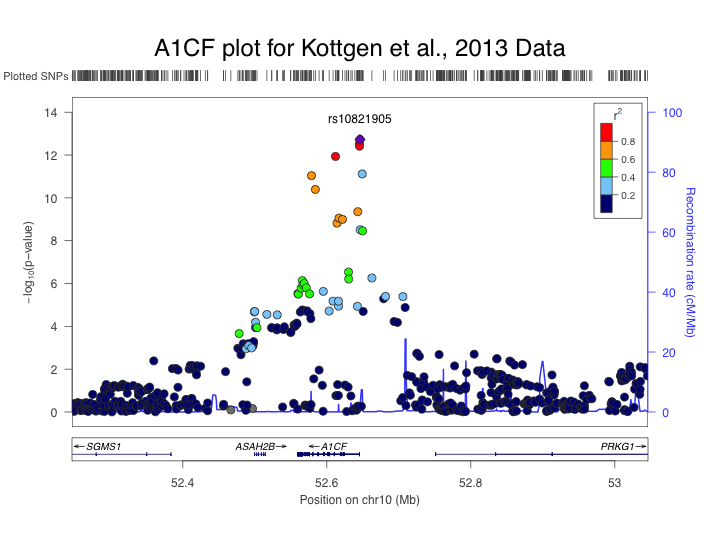 | 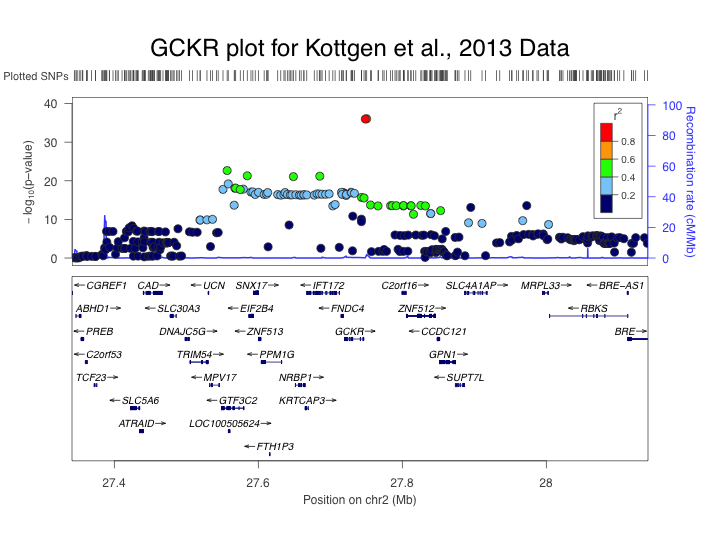 |
| --- | --- |
| A) | B) |

Figure S2: LocusZoom pictures of regional association of A1CF and GCKR in Europeans in the study by Köttgen et al., (2013). A) The *A1CF* association signal defines a single causal gene of high-prior probability. B) Multiple genes underlying a serum urate association signal at the *GCKR* locus.
